# Difficult Cases of Labor

**Published:** 1890-06

**Authors:** Robert W. Westmoreland

**Affiliations:** Atlanta, Ga.


### DIFFICULT CASES OF LABOR.

BY ROBERT W. WESTMORELAND, M. D., ATLANTA, GA.

The general experience will bear me out in the assertion that it requires but little learning or experience in the successful conduction of the ordinary case of labor; the encouragement of the patient, tying the chord and delivery of the placenta comprising about the whole of the duties, ordinarily, of the accoucheur.

Could it be accurately determined when these *benign* labors were to occur, the midwife could be safely employed by those of limited means, and this economy in fees be judiciously indulged. But here Fate favors the doctor financially. It cannot be known, by the uninitiated, when grave complications will occur, where prompt and experienced action is imperative to the salvation of life.

I venture to recount some of the limited experience which has occurred to me in this line, with the hope that something in the narrative may interest some one.

I was called hurriedly one afternoon in 1889 to see Mrs. B. She had been in active labor two days and a night. On my arrival I found three other doctors present, and a room full of friends of the patient. Every one seemed to be fully alarmed at the prospect of impending death, and truly the woman had the appearance of being *in extremis*. An examination revealed a lateral position, with an arm presentation, One arm had been torn entirely off in efforts at forcible delivery.

On arising from the examination I was promptly met by the assurance from the other attendants, that they did not see how the delivery was to be accomplished without dissection of the fœtus. This, truly, would have done as well, as far as the child's life was concerned, but, in view of the imperilled state of the woman's life, I could not consider it judicious.

I, at once, therefore, proceeded to put her under the influence of chloroform, despite the protestations of the others concerned. So much was the anæsthetic feared, that I experienced the greatest difficulty in effecting complete anæsthesia, by those administering the drug secretly holding it off. When she was thoroughly under the influence, I introduced my hand, with little difficulty, grasped the feet, and, turning easily, delivered the dead fœtus in a very few minutes. This was the most difficult part, then, of the labor, as the afterbirth was no trouble, and no more than usual hemorrhage supervened. The woman made a remarkably quick recovery. I believe, fully, if this course had been pursued earlier, not only would the mother have been saved much trouble and suffering, but the child's life have been saved as well.

In August, 1888, I was called in consultation to see a woman in labor with the last of twins. The first had been delivered with little difficulty, but every effort had failed to produce the last. After a day and night's trial I was called in. I found a lateral position with the back presenting. Turning was accomplished under thorough anaesthesia, and a dead child was promptly delivered. The woman succumbed from exhaustion, the following day.

In Feb'y. 1889, I attended Mrs. P. in a case of labor. The position and presentation was as good as could be desired. But after two days and two nights, active labor, and very little headway made, I anesthetized thoroughly, turned and delivered, after a great deal of difficulty, a dead child. Here the child was too large to be delivered by the natural pains, and would have been killed by the force necessary to deliver by forceps, or any other means. The patient suffered considerably from puerperal fever, but made a perfect recovery.

The lesson I learned from this experience has led me to turn in all such cases, in preference to the use of forceps or other means of delivery. The abnormal position or size is such as nature cannot cope with alone. Delay jeopardizes the life, not only of the child, but the mother as well, when promptness will often save both. In the hands of the expert, delivery by the breech (as the result of the turning) is but very little less safe than by the vertex. Therefore, I think, it is a safe plan to turn and deliver in cases that promise to be difficult and dangerously protracted.

---

*Merck's Bulletin*, April, 1890, recalls two or three excellent and soon to be seasonable formulæ, published two years ago, or longer, in *The Practitioner*, the strength of the ingredients being adapted to the U. S. Ph :—

IN INFANT'S "SUMMER COMPLAINT:" Tincture Indian cannabis, twenty-four drops; spirit chloroform, five drops; tincture kino, 3.40 grammes [1 fl. dr.]; peppermint-water, to make 26.25 grammes [7 fl. dr.]; add, distilled water, 3.75 grammes [1 fl. dr.]. Shake well—teaspoonful every one or two or three hours.

IN ADULTS' INTENSIVE LONG-STANDING DIARRHŒA: Tincture Indian cannabis, twenty-four drops; solution morphine bimeconate (1:80), five to ten drops; spirit ammonia aromatic, twenty drops; spirit cloroform, ten drops; distilled water, to make 30 grammes [ 1 fl. oz.]. Two tablespoonfuls every one or two or three hours—strict fast during the next few hours—only a little rum-and-water.

IN DYSPEPTIC DIARRHŒA: Tincture Indian cannabis, twenty-four to forty-eight drops ; bismuth sub-nitrate, 0.6 gramme [9 grains ]; mucilage acacia, 18.75 grammes [4 1-2 fl. dr.], spirit chloroform, ten drops ; peppermint-water, 26.25 grammes [7 fl. dr.]. Shake well—tablespoonful (or more) before or after meals—large doses are best taken after meals.

